# The impact of symptoms on quality of life before and after diagnosis of coeliac disease: the results from a Polish population survey and comparison with the results from the United Kingdom

**DOI:** 10.1186/s12876-021-01673-0

**Published:** 2021-03-04

**Authors:** Emilia Majsiak, Magdalena Choina, Dominik Golicki, Alastair M. Gray, Bożena Cukrowska

**Affiliations:** 1 Faculty of Medicine, Collegium Medicum, Cardinal Stefan Wyszyński University in Warsaw, Auditorium Maximum, bldg. 21, room 201 (II floor), st. Kazimierza Wóycickiego 1/3, 01-938 Warsaw, Poland; 2 Polish-Ukrainian Foundation of Medicine Development, Lublin, Poland; 3 Department of Experimental and Clinical Pharmacology, Medical University of Warsaw, Warsaw, Poland; 4 Health Economics Research Centre, Nuffield Department of Population Health, University of Oxford, Oxford, UK; 5 Department of Pathology, Children’s Memorial Health Institute, Warsaw, Poland

**Keywords:** Coeliac disease, Quality of life, Diagnostic process

## Abstract

**Background:**

Coeliac disease (CD) is characterised by diverse clinical symptoms, which may cause diagnostic problems and reduce the patients’ quality of life. A study conducted in the United Kingdom (UK) revealed that the mean time between the onset of coeliac symptoms and being diagnosed was above 13 years. This study aimed to analyse the diagnostic process of CD in Poland and evaluate the quality of life of patients before and after CD diagnosis. In addition, results were compared to the results of the original study conducted in the UK.

**Methods:**

The study included 2500 members of the Polish Coeliac Society. The patients were asked to complete a questionnaire containing questions on socio-demographic factors, clinical aspects and quality of life, using the EQ-5D questionnaire. Questionnaires received from 796 respondents were included in the final analysis.

**Results:**

The most common symptoms reported by respondents were bloating (75%), abdominal pain (72%), chronic fatigue (63%) and anaemia (58%). Anaemia was the most persistent symptom, with mean duration prior to CD diagnosis of 9.2 years, whereas diarrhoea was observed for the shortest period (4.7 years). The mean duration of any symptom before CD diagnosis was 7.3 years, compared to 13.2 years in the UK. CD diagnosis and the introduction of a gluten-free diet substantially improved the quality of life in each of the five EQ-5D-5L health dimensions: pain and discomfort, anxiety and depression, usual activities, self-care and mobility (*p* < 0.001), the EQ-Index by 0.149 (SD 0.23) and the EQ-VAS by 30.4 (SD 28.3) points.

**Conclusions:**

Duration of symptoms prior to the diagnosis of CD in Poland, although shorter than in the UK, was long with an average of 7.3 years from first CD symptoms. Faster CD diagnosis after the onset of symptoms in Polish respondents may be related to a higher percentage of children in the Polish sample. Introduction of a gluten-free diet improves coeliac patients’ quality of life. These results suggest that doctors should be made more aware of CD and its symptoms across all age groups.

## Background

Coeliac disease (CD) is considered to be one of the most common disorders of the alimentary tract. It is estimated that CD affects around 1% of people worldwide and its incidence is increasing [1]. Despite new diagnostic tools, many cases of CD remain undiagnosed [2, 3], which results from the fact that CD often manifests with a wide range of symptoms, many of which are not specific to CD, or its course can even be asymptomatic [4]. Due to concomitant symptoms, CD may be categorised as classical, non-classical, potential or sub-clinical [5]. Symptoms of classical CD are mainly gastrointestinal and include abdominal pain, nausea, vomiting, bloating, and diarrhoea [6]. In the non-classical type of CD symptoms are extra-intestinal, e. g. anaemia, decreased bone density or impaired growth. It should be emphasised that the extra-intestinal symptoms have been believed for a long time to be the consequence of disturbed absorption of nutrients [7–9]. Patients with non-classical CD also suffer from headaches, chronic fatigue, depression and skin diseases [10]. Many researchers have suggested that more and more patients who are diagnosed with CD, especially older children and adults, suffer mainly from extra-intestinal symptoms [11]. It has not been established whether this results from a true increasing incidence of non-classical CD, or from greater awareness of physicians and CD patients [12], or from more effective detection of CD in adults in recent years.

A gluten-free diet (GFD) appears to be the most effective factor in the treatment of CD. Early detection of CD and the introduction of a GFD can improve the quality of life of CD patients and reduce expenses arising from the diagnostic process and subsequent therapy [13].

However, the study conducted by Gray and Papanicolas reported that in the United Kingdom (UK) the mean time between the onset of CD symptoms and establishing the diagnosis was over 13 years [14]. That finding, and suspicion that in Poland the mean time between the onset of coeliac symptoms and CD diagnosis could be even longer than in the UK led us to conduct a similar study using comparable instruments and methods among Polish CD patients.

## Methods

### Aim

The aim of the study was to analyse the diagnostic process of CD in Poland and the quality of life of respondents before and after CD diagnosis. The results were compared with the results of the study carried out in the UK.

### Respondents

Two and a half thousand respondents, who were members of the Polish Coeliac Society were enrolled in the study. They were sent a letter with information on the planned study and a request to complete the attached questionnaire. Of this number, 969 (38.76%) surveyees returned the questionnaire. Answerers (n = 173) who had adopted a GFD on their own, which entitled them to become members of the Society, were excluded from the study. Finally, 796 questionnaires were included in the analysis. If respondents were under the age of 18, their parents or guardians were asked to complete the questionnaire.

The Bioethics Committee of the Children's Memorial Health Institute gave its consent for the study to be conducted (No. 48 /KBE/2017).

### Questionnaires

The original British questionnaire “The Impact of Coeliac Disease on Your Life: A Survey of Your Views” was used in this study with the consent of the authors [15]. The questionnaire consisted of two parts. In the first part, the British authors included 44 questions on socio-demographic factors, clinical aspects and CD-related costs. In the second part they distributed a generic quality of life questionnaire—the three level EQ-5D (EQ-5D-3L) [16]. Due to different economic conditions in the two countries, some questions needed modifications. In addition, as members of the Polish Coeliac Society are both patients who were diagnosed with CD and people who introduced GFD themselves the questionnaire distributed among them needed some modification compared to the original questionnaire from the British study. In order to verify how the CD diagnosis had been established, a question about the introduction of GFD was added. The Polish respondents could choose between two options: the first one was “On your own, without any examination” and the second one was “After establishing the diagnosis of coeliac disease”. If the surveyee chose the first answer, they finished completing the questionnaire and they were automatically excluded from further statistical analysis. The surveyees who chose the other option, were then asked about the method in which the diagnosis was made—if they underwent blood test (without specification of the serological tests), duodenal biopsy, genetic examination or any other type of examination. If the respondent had undergone any other type of examination, they had to specify the examination. Next question that verified the method in which CD was diagnosed concerned the specialization of clinician who had made CD diagnosis.

The first part of the original questionnaire was translated into Polish, and in order to check the quality of the translated versions, the documents were then back-translated from Polish into English and compared with the original version of the questionnaire. There were no significant differences.

In the Polish study, the new five-level version of the EQ-5D (EQ-5D-5L) was used instead of the three level version [17]. The EQ-5D questionnaire consists of two parts: a descriptive system and a visual analogue scale (EQ VAS). In the first part, surveyees are asked to self-assess their health in five dimensions: mobility, self-care, usual activities, pain and discomfort, anxiety and depression, using a 5-level scale of “no problems”, “slight problems”, “moderate problems”, “severe problems” or “extreme problems/lack of ability”. In the Polish survey, as in the UK, respondents were asked to assess their quality of life at the time of their diagnosis and at the time of the survey. In order to compare the results of the UK and Polish surveys (measured on a 3-level and 5-level scale, respectively), we dichotomised the answers into “no problems” and “any problems” groups. The results of Polish coeliac respondents before and after diagnosis were compared. Additional comparison with age- and sex-adjusted Polish normative data was performed [18]. Based on the descriptive system results and Polish directly measured EQ-5D-5L value set, EQ-Index was calculated (single fraction representing quality of life, anchored at “0”—death and “1”—full health) [19]. In the second part of the EQ-5D questionnaire the Polish CD answerers evaluated the quality of their life before and after CD diagnosis on a visual analogue scale from 0 to 100 points, where 0 and 100 stand for worst and best imaginable health, respectively. The results of EQ VAS were also compared with Polish population normative data [20].

### Statistical analysis

Statistical comparisons for two variables were made using Wilcoxon’s test, while comparisons for three variables was made using Kruskal–Wallis’ test. As long as Kruskal–Wallis’ test indicates only the presence of statistically significant difference, further analysis was made using Bonferroni’s test. In order to determine statistically significant correlation between qualitative variables, Chi-squared test was used. Spearman’s rank correlation coefficient was used to report correlation between variables. Continuous variables was measured by mean values, whereas variability around mean values was reported in terms of standard deviations. The precision around mean values was described with 95% confidence intervals. Statistical analysis was done using Statistica 10 (StatSoft Polska).

## Results

### Characteristics of the respondents

A similar number of respondents took part in both studies: in the UK the study group consisted of 788 CD patients, compared to 796 patients in Poland (Table 1). Among the Polish respondents there were 642 (80.7%) females and 154 (19.3%) males, whereas among the British respondents there were 557 (72%) women. The mean age of the surveyees in Poland was 29.31 years, while in the UK it was 52 years, a statistically significant difference (*p* < 0.001). Children constituted 28.1% (n = 224) of the whole Polish group, but in the British group children made up only 12% (n = 97). In the British study group 10% (n = 80) of respondents were aged 65 or over, whereas only 2.6% (n = 21) of the Polish group was aged 65 or over. It cannot be excluded that the Polish respondents are in some way unrepresentative of the entire group of Polish patients diagnosed with CD.

**Table 1:** Characteristics of the Polish and the British study groups

Variables	Poland	The United Kingdom
No. sampled	2500	2000
No. of completed and returned questionnaires	796	788
Response rate	38.76%	39.4%
Sex—no. (%)		
Female	642 (80.7)	559 (70.9)
Male	154 (19.3)	224 (28.4)
Average age at survey—mean (SD)	29.31 (N/A)	52 (19)
Average age at diagnosis—mean (SD)	24.1 (15.9)	41 (19)
Age at diagnosis—no. (%)		
< 18	224 (28.1%)	97 (12.4%)
18–34	262 (32.9%)	134 (17.2%)
35–44	198 (24.9%)	188 (24.1%)
45–54	61 (7.7%)	153 (19.6%)
55–64	30 (3.8%)	128 (16.4%)
> = 65	21 (2.6%)	80 (10.3%)
Family members with diagnosed CD—no. (%)		
Respondent only person	627 (78.8)	728 (93)
One or more family members diagnosed with CD	169 (21.2)	55 (7)
Following GFD—no. (%)		
All the time	746 (93.7)	N/A^1^
Most of the time	46 (5.8)	N/A
Some/little/none of the time	3 (0.4)	N/A

*N/A* not available

CD diagnosis in Polish patients was always done by medical doctors, and in adults were based on blood tests and/or duodenal biopsy examination. All Polish children underwent serological tests, and according to ESPGHAN guidelines [6] one third of them was diagnosed without duodenal biopsy. Others underwent small intestinal biopsy.

Of the total number of the Polish interviewees, 627 (78.77%) CD respondents were the only family member of their household with diagnosed CD, while the disease had been diagnosed in another different family member in 169 (21.23%) cases. In the UK, 728 (93%) respondents were the only family member of their household with CD and 7% of the British surveyees shared their household with another family member diagnosed with CD.

The mean age for CD diagnosis in the Polish study group was 24.1 years in contrast to the UK, where CD was diagnosed on average at the age of 41.3. The difference in the mean age at CD diagnosis between the two countries was 17 years (*p* < 0.001). The mean age for CD diagnosis in Poland before the year 2000 was 9.4 years, and for respondents diagnosed after the year 2000 the mean age was 25.1 years, whereas in the UK these values were 39 and 44 years respectively. The difference in the mean age at diagnosis in Poland before and after 2000 was 15.7 years (*p* < 0.001) and in the UK was five years (*p* < 0.001). In Poland, 49 (6.2%) CD patients were diagnosed before the year 2000, while in the UK 54% of surveyees had been diagnosed before that year.

The difference in the mean age of CD detection between Poland and the UK before the year 2000 was 30 years (*p* < 0.001), but after the year 2000 it was 18.9 years (*p* < 0.001).

#### Clinical symptoms

Respondents were asked to report the symptoms they had experienced and their mean duration before CD diagnosis. The most common CD symptoms reported by Polish surveyees included: flatulence (75%), abdominal pain/bloating (72%), chronic fatigue (63%) and anaemia (58%). The British respondents suffered from bloating (71%), diarrhoea (70%), anaemia (65%), chronic fatigue (62%) and weight loss (61%) most frequently. In the Polish study group, 664 (83.41%) respondents reported at least four symptoms before CD diagnosis and the result was similar in the UK (78%). Both in Poland and in the UK only 1% of the respondents did not report any symptom before diagnosis (Table 2).

**Table 2:** Incidence and the mean duration of symptoms before CD diagnosis in Polish and British respondents

Symptoms	Poland					The United Kingdom				
	Number (%) of reported symptoms (796 respondents)			Duration in years		Number (%) of reported symptoms (777 respondents)			Duration in years	
	Number	%	(95% CI)	Mean	(95% CI)	Number	%	(95% CI)	Mean	(95% CI)
Any symptom	788	99	(98, 100)	6.4	(5.9, 6.8)	771	99	(98, 100)	13.2	(12.2, 14.4)
Flatulence	594	75	(72, 78)	8.1	(7.2, 9.0)	368	47	(43, 50)	9.5	(8.6, 10.4)
Abdominal pain/Bloating	574	72	(69, 75)	7.1	(6.3, 7.8)	556	71	(67, 74)	7.9	(7.0, 8.7)
Chronic fatigue	505	63	(60, 67)	6.3	(5.6, 6.9)	488	62	(59, 60)	7.1	(6.3, 7.9)
Anaemia	459	58	(54, 61)	9.2	(8.2, 10.2)	509	65	(61, 68)	11.5	(10.5, 12.6)
Diarrhoea	449	56	(53, 60)	4.7	(4.0, 5.4)	553	70	(67, 73)	6.9	(6.1, 7.8)
Headache	366	46	(43, 49)	8.6	(7.6, 9.6)	232	30	(26, 33)	10.3	(9.3, 11.3)
Weight loss	349	44	(40, 47)	5.2	(4.3, 6.1)	349	44	(40, 47)	5.2	(4.3, 6.1)
Skin rash	301	38	(34, 41)	7.4	(6.2, 8.5)	301	38	(34, 41)	7.4	(6.2, 8.5)
Joint pains	280	35	(32, 39)	7.6	(6.6, 8.7)	220	28	(25, 31)	8.2	(7.4, 9.0)
Constipation	276	35	(31, 38)	8.5	(7.2, 9.7)	207	26	(23, 29)	12.6	(11.5, 13.6)
Mouth ulcer	272	34	(31, 38)	7.2	(6.0, 8.3)	236	30	(27, 33)	11.2	(10.1, 12.3)
Depression	153	19	(17, 22)	5.8	(4.6, 7.0)	185	24	(21, 27)	9.2	(8.3, 10.1)
Ataxia	60	8	(6, 10)	5.6	(3.5, 7.7)	39	5	(3, 7)	6.1	(5.2, 6.9)
Osteoporosis	57	7	(6, 9)	6.5	(4.7, 8.3)	91	12	(10, 14)	7.7	(6.7, 8.6)
No symptoms	8	1	(0, 2)			6	1	(0, 2)		

The mean duration of symptoms before diagnosis in Poland ranged from 4.7 to 9.2 years and depended on the particular symptom. The mean duration of any symptom before establishing the diagnosis was 7.3 years for the whole analysed group and 9 years for the adult respondents. In surveyees with CD diagnosed before 2000 the mean duration of symptoms before CD detection was 6.4 years, and in interviewees with CD diagnosed after 2000 the mean duration was 7.3 years (Table 3). There was a statistically significant difference (*p* < 0.0.01) between the mean duration of any symptom in Poland and the UK (7.3 years and 13.2 years, respectively). A statistically significant difference (*p* < 0.001) was also observed for symptoms that lasted for the longest time. In Poland it was anaemia (9.2 years), and in the UK was constipation (12.6 years). Respondents in Poland suffered for the shortest time from diarrhoea (4.7 years), compared to weight loss (5.5 years) in the UK. It should be highlighted that the mean duration of symptoms in British surveyees fell from 14.5 years in those diagnosed before the year 2000 to 12 years in respondents diagnosed after the year 2000 (*p* < 0.001).

**Table 3:** The mean duration of symptoms before diagnosis of CD in 796 Polish respondents

	Year of diagnosis					
	In total		≤ 2000		> 2000	
	Mean	SD	Mean	SD	Mean	SD
Diarrhoea	4.7	7.52	3.8	6.46	4.7	7.60
Constipation	8.5	10.45	7.9	11.32	8.5	10.44
Chronic fatigue	6.3	7.51	8.5	11.83	6.1	7.19
Abdominal pain	7.1	9.16	6.1	8.56	7.1	9.21
Intestinal gases/bloating	8.1	9.79	7.3	9.51	8.2	9.82
Headaches	8.6	9.56	5.2	6.79	8.8	9.65
Joint pains	7.6	8.63	11.8	8.62	7.4	8.60
Osteoporosis	6.5	6.50	3.7	5.48	6.7	6.56
Skin rash	7.4	9.88	6.4	9.27	7.4	9.93
Mouth ulcer/aphtae/lip sores	7.2	9.36	4.1	5.03	7.4	9.59
Anaemia	9.2	10.86	7.2	9.05	9.3	10.96
Depression	5.8	7.38	5.9	5.16	5.8	7.47
Ataxia/ impaired coordination of muscle movements	5.6	8.10	3.9	3.78	5.7	8.28
Weight loss (≥ 10%)	5.2	8.41	5.2	6.87	5.2	8.56
Fatigue	6.6	7.97	7.8	10.90	6.6	7.80

#### Diagnostic process

Information on the number of appointments with General Practitioners (GPs) due to symptoms which occurred before the diagnosis of CD was provided by 779 (97.9%) Polish respondents and 655 (83%) British surveyees. The respondents from Poland made more pre-diagnosis medical appointments (17.8 on average) than the British ones (13 on average). The Polish interviewees in whom the disease was diagnosed before 2000, made on average 21.5 appointments; with regards to the British answerers, the number of appointments exceeded 17. In both countries, the authors noted a decrease in the average number of appointments with GPs in respondents diagnosed after 2000 (17.5 appointments in Poland, seven appointments in the UK). Both Polish and British results implied the relationship between the duration of symptoms and the number of appointments with GPs: the longer the duration of symptoms, the greater the number of appointments (*p* < 0.001) (Table 4).

**Table 4:** Number of GPs consultations pre-diagnosis about symptoms among Polish CD patients, by duration of symptoms

Duration of symptoms before the diagnosis in years	Number of appointments		
	Number	Mean	95% CI
< 1	94	7.6	(5.3, 9.8)
1–5	367	13.5	(11.7, 15.3)
5–10	172	19.5	(16.0, 23.0)
10–20	116	29.4	(24.0, 34.9)
> 20	44	38.0	(24.5, 51.5)
In total	793	17.8	(16.1, 19.4)

#### Quality of life according to EQ-5D questionnaire

Almost all Polish respondents completed the EQ-5D quality of life questionnaire (n = 790, 99.2%). The responses on each of the five dimensions of the EQ-5D were compared both with standard values for the general Polish population and the UK CD surveyees (Table 5). Patients with CD before diagnosis, differed significantly (*p* < 0.001) from age-adjusted Polish normative population in terms of all five EQ-5D dimensions. The most significant differences involved pain/discomfort, anxiety/depression and usual activities dimensions (43%, 43% and 33% differences, respectively). The diagnosis and introduction of GFD resulted in significant quality of life (QoL) amelioration within all dimensions, with the size of gain proportional to previous quality of life deterioration. Despite improvement, CD respondents still differed from age-adjusted normative data within all dimensions, except self-care (*p* = 0.08).

**Table 5:** Frequency of “No problems” answers according to EQ-5D-5L: results in Polish and British coeliac respondents

	Poland								United Kingdom				
	Pre-diagnosis (N = 790)		Post-diagnosis (N = 790)		Polish population norm^a^	*p* value pre-diagnosis vs population norm^b^	*p* value post-diagnosis vs population norm^b^	*p* value pre-diagnosis vs post-diagnosis^b^	Pre-diagnosis (N = 697)	Post-diagnosis (N = 697)	UK population norm^c^	*p* value pre-diagnosis UK versus Poland^b^	*p* value post-diagnosis UK versus Poland^b^
	n	%	n	%	%				%	%	%		
Mobility	614	78	666	84	91	< .001	< .001	< .001	75	86	78	NS	NS
Self-care	695	88	759	96	97	< .001	0.08	< .01	93	97	93	< .002	NS
Usual activities	478	61	602	76	94	< .001	< .001	< .001	58	82	78	NS	< 0.01
Pain/ discomfort	189	24	399	51	67	< .001	< .001	< .001	22	60	58	NS	< 0.001
Anxiety/ depression	211	27	377	48	70	< .001	< .001	< .001	50	74	75	< .001	< 0.001

*NS* not significant

^a^Polish population norms from Golicki and Niewada 2017 study [18], standardized to age distribution of coeliac disease respondents in current study during survey

^b^Fisher’s exact test

^c^UK population norms from Health Survey for England 1996, standardized to age distribution of Coeliac UK survey respondents at the time of survey

In comparison to UK respondents, Polish answerers had before CD diagnosis more problems with anxiety and depression (*p* < 0.001) and self-care (*p* < 0.002). After the diagnosis and introduction of GFD, Polish CD respondents differed from their UK counterparts within anxiety/depression, pain/discomfort and usual activities dimensions.

EQ-Index, based on Polish directly measured EQ-5D-5L value set, is an objective tool to assess the quality of life, where “0” means death and “1” means full health. Among the Polish respondents, the mean quality of life before the diagnosis was 0.792 (SD 0.239; Table 6). After the diagnosis and introduction of GFD it rose to 0.941 (SD 0.105; Wilcoxon's signed ranks test: *p* < 0.001). While Polish surveyees experienced an EQ-Index improvement of 0.149, their UK counterparts noticed even more significant increase (of 0.27; *p* < 0.001).

**Table 6:** EQ-Index and EQ-VAS in Polish coeliac respondents, in UK coeliac respondents and in Polish population

	Polish coeliac respondents		Polish population norm^a^		UK coeliac respondents	
	Mean	95% CI	Mean	95% CI	Mean	95% CI
EQ-5D Index^b^						
pre-diagnosis	0.792	(0.809, 0.775)	0.971	(0.969, 0.972)	0.56	(0.54, 0.59)
post-diagnosis	0.941	(0.933, 0.948)	0.965	(0.963, 0.967)	0.84	(0.82, 0.85)
change	0.149	(0.133, 0.165)			0.27	(0.25, 0.30)
EQ-VAS						
pre-diagnosis	44.7	(43.0, 46.4)	82.7	(82.4, 83.0)	47	(45, 49)
post-diagnosis	75.1	(73.8, 76.4)	81.6	(81.2, 82.0)	79	(78, 80)
change	30.4	(28.4, 32.4)			32	nnn

^a^Polish population norms from Golicki and Niewada [18] (EQ-5D-5L Index) and Golicki and Niewada [20] (EQ-VAS) studies

^b^Polish index estimated with Polish EQ-5D-5L TTO/DCE-based value set, UK index estimated with UK EQ-5D-3L TTO-based value set

Similarly, in terms of EQ VAS results, Polish CD respondents experienced significant amelioration of QoL after the diagnosis and introduction of GFD – by 30.4 (SD 28.3; Wilcoxon's signed ranks test: *p* < 0.001; Table 6) points. Subjective perception of QoL before the diagnosis, which was significantly different from the age- and sex-adjusted population norm (44.7 and 82.7 points, respectively, *p* < 0.001), approached the population norm after the diagnosis, but remained significantly different (75.1 and 81.6; *p* < 0.001).

## Discussion

The study conducted by Gray and Papanicolas confirmed that CD poses diagnostic problems, with a mean time between the onset of coeliac symptoms and diagnosis of 13 years [15]. Those results met with some disbelief among Polish clinicians, which encouraged the authors of this study to conduct a similar analysis in Poland, using a similar questionnaire and methods.

Based on the responses to the questionnaire, a similar number of surveyees was admitted to the study in Poland and in the UK. In both countries, females were the majority of the study group. This discrepancy in occurrence of CD between sexes has been already observed.

The mean age of respondents was significantly higher in the UK than in Poland. This could indicate that CD in adults may be diagnosed less frequently in Poland than in the UK. Another aspect that may have influenced the results could be the difference in the number of children and adults who took part in each study. This, in turn, could reflect differences in the age composition of those participating in CD associations, for whatever reason. However, these results suggest that Polish physicians typically deal with CD as a paediatric disorder, and have more limited experience of diagnosing CD in older adults. Until the 1980s CD was considered a rare disorder, affecting mostly children. Serological screening, implemented in the 1990s, revealed that CD could affect people of all ages [21, 22]. In one Polish manual, called “Internal diseases: a manual for students”, edited by Franciszek Kokot and issued in 1991, we can read “Since coeliac disease is a paediatric disorder, it will not be presented in details in this manual” [23]. In the 6th issue of the same book from the year 1996, on page 250, it is stated that “(CD) disease will not be presented in this book since it is a paediatric disorder” [24]. This implies that in Poland in the 1990s, the CD was still regarded as a paediatric disease, and this would be consistent with the pattern of diagnosis observed in this study, in contrast to the results from the British survey. The increase in the mean age at diagnosis in Poland and in the UK corresponds to world tendencies—CD is more and more often diagnosed in adults and in elderly [25]. Interestingly, in Poland the difference in the mean age of answerers diagnosed before and after 2000 was much more significant than in the UK (almost 16 years compared to 5 years, respectively). These data imply that physicians in Poland are increasingly identifying CD as not just a paediatric disorder, but that further efforts to improve the diagnosis process in adults should be made.

Although many authors have noted the increasing incidence of non-classical CD with symptoms outside the gastrointestinal tract [12], respondents in Poland and in the UK reported that bloating and abdominal pain were the most common symptoms. Other commonly reported CD symptoms both in Poland and in the UK included anaemia and fatigue. The vast majority of respondents from both countries were polysymptomatic (80% in the UK and almost 85% in Poland). However, only 1% of respondents from both countries were asymptomatic prior to CD diagnosis.

The results of both Polish and British study indicated that coeliac symptoms typically precede a formal diagnosis by many years. The mean duration of any symptom before CD detection was shorter in Poland (more than seven years) than in the UK (more than thirteen years). A more recent study by Gray and Violato published in May 2019 revealed that the diagnostic process shortened in the UK to 12.8 years, but the reduction was not significant in comparison to the findings based on the survey from 2006 [26]. However, whereas the mean duration of any symptom among British respondents was shorter in those diagnosed after the year 2000 compared with those diagnosed before 2000, the tendency in Poland was the opposite: before 2000, the mean duration of symptoms was 6.4 years; after 2000 it increased to 7.3 years. This could be attributed to changes in the health care system, e.g. implementation of referrals to specialists, including gastroenterologists, and greater detection of CD in adult surveyees.

Lack of proper diagnosis and the presence of CD symptoms affecting the quality of life result in respondents making regular appointments with GPs. The results of the study showed that Polish CD surveyees (almost 98% of respondents) sought help in primary health care clinics more frequently than British ones (83% of respondents), and on average made more pre-diagnosis medical appointments (on average, almost 18) than the British interviewees (on average, 13). In both countries a decrease was observed in the mean number of appointments with GPs among respondents diagnosed with CD after the year 2000 in comparison to those diagnosed before 2000. However, Polish surveyees diagnosed after 2000 sought help in primary health care clinics 2.5 times more often than British answerers. The Polish and the British results implied a relationship between the duration of symptoms and the number of appointments with GPs. These data indicate that GPs should be reminded about CD in order to decrease the number of appointments before the diagnosis of CD. Gastroenterologists could provide family doctors with guidelines regarding patients suspected of CD. These guidelines might indicate which patients should undergo serological examinations and be referred to a specialist. This could make the diagnosis process quicker, with fewer appointments, a more rapid introduction of GFD, and lower costs related to diagnosing CD and treating patients affected by this disease.

Many studies look at the QoL in patients with CD. Most of them are based on the use of generic questionnaires, especially Medical Outcomes Study Short Form-36 (SF-36) [13]. Definitely, SF-36 is the most widely used quality of life questionnaire from the category of health profiles. In our study, we decided to use the EQ-5D. The EQ-5D questionnaire is the most frequently used tool for measuring health state utility values (patient preferences), a tool for quality-adjusted life years (QALY) estimation and a key to conduct cost-effectiveness analysis. Majority of health technology assessment agencies, including the National Institute of Health and Clinical Excellence in England, recommend the EQ-5D [27]. Our study is the first to use a new five-level version of the EQ-5D (namely EQ-5D-5L) in CD patients [16]. We acknowledge that other generic and disease-specific instruments are also widely used in gastroenterology, and future research would be valuable to establish the relationship between these, for example by means of mapping studies.

Both in Poland and in the UK, quality of life in the five studied dimensions was significantly higher after CD diagnosis than before it. In contrast to the British respondents, the Polish CD respondents more often suffered from pain and discomfort after diagnosis than the general population, and from more anxiety and depression both before and after diagnosis. These differences might result from a different socio-economic status in both countries. The UK has implemented different solutions, regarding the health care of CD patients: probably more time is devoted to them and their disease is treated more seriously. Respondents’ attitude towards the disease could also be crucial.

Early detection of CD and elimination of gluten products from the diet might reduce symptoms of the disease and improve the patients’ quality of life [10]. A meta-analysis published in 2017 showed that GFD significantly improved health-related QoL in adults with CD [13]. The British respondents rated the quality of their life better than the Polish ones both before and after CD diagnosis. Neither of the studies revealed a relationship between the self-rated quality of life before and after the diagnosis and the duration of symptoms. Both studies, the Polish and the British, showed that the value of the mean quality of life of respondents with CD is substantially higher after diagnosis. The authors of the study decided not to compare the mean quality of life of the surveyees diagnosed before and after the year 2000 in both countries in view of disproportions in the groups. While in the UK 54% of CD respondents were diagnosed before the year 2000, in Poland slightly more than 6% of the answerers were diagnosed at that time.

We are aware of some limitations of our study. The assessment of diagnostic process of CD as well as of QoL before and after CD diagnosis was done retrospectively, so it is susceptible to response bias. However, retrospective analysis is inevitable because there are no large long-term prospective studies, during which data is collected frequently. Retrospective assessment is a method that has been widely used in studies on CD and on other diseases [28–30] but we cannot guarantee that the results of analogous prospective studies would be similar. We also note a 10-year gap between the results of the original British study and the outcomes of the Polish analysis: this may restrict the comparison, especially given that CD diagnostic procedures have been changing over time. However, despite the time difference, the diagnostic process of CD in Poland, although shorter than in the UK, is still very long. This should be a matter of importance not only for the patients who have not been diagnosed with CD yet, but for the whole society, as they bear the cost of numerous appointments with doctors and of absence at work or at school. As the study aimed to include only members of Polish Coeliac Society with clinically confirmed CD, those who introduced GFD on their own were automatically excluded from further analysis. We are cognizant of the disproportion between children and adults in study groups in the UK and in Poland—whereas in the UK slightly more than 10% of participant was underage, in Poland more than a quarter of respondents was less than 18 years old. This discrepancy may illustrate the common attitude in Poland, where CD is still regarded as paediatric disease. In order to verify how the manner of diagnosing with CD children and adults in Poland has changed over the years, another analysis, comparing the results from these two age groups, should be performed.

A one of last study done by Choung et al. Effect of a Gluten-free Diet on Quality of Life in Patients With Nonclassical Versus Classical Presentations of Celiac Disease” shown a differences between patients with classical and nonclassical presentation [31]. The authors indicate that such an analysis of the studied population would have been a useful and could be a possible future work.

We are conscious that the sample drawn from Polish Coeliac Society may not be fully representative of the entire CD population in Poland. However, there is no current data on epidemiology of CD in Poland. In spite of the possible sampling bias, the improvement in QoL after CD diagnosis is significant.

## Conclusions

The results indicate that the mean time between the onset of coeliac symptoms and making a diagnosis is shorter in Poland than in the UK, partly because it is more focused on diagnosing paediatric respondents (particularly before the year 2000). More awareness among doctors of CD as an adult disease may alter this pattern in future.

The diagnosis followed by GFD implementation substantially improves the quality of life of CD patients. Hence, in order to shorten the diagnostic process and promptly initiate a therapy, anything which can reduce the delays in diagnosis, including familiarising GPs and medical specialists with symptoms accompanying this disease in adults, would be beneficial.

## Data Availability

The datasets generated and/or analysed during the current study are not publicly available because they are also being used for further ongoing analyses, but are available from the corresponding author on reasonable request.

## Abbreviations

CD: Coeliac disease
GFD: Gluten-free diet
SD: Standard deviation
N/A: Not available
GPs: General practitioners
QoL: Quality of life
NS: Not significant
